# USP1, an MAPK Target with Chaperone-like Activity, Regulates Innate Plant Immunity in *Arabidopsis*

**DOI:** 10.3390/ijms27219426

**Published:** 2026-10-22

**Authors:** Prabhu Manickam, Hanna Alhoraibi, Alaguraj Veluchamy, Marilia Almeida-Trapp, Naganand Rayapuram, Heribert Hirt

**Affiliations:** 1 Center for Desert Agriculture, King Abdullah University of Science and Technology (KAUST), Thuwal 23955-6900, Saudi Arabia; 2 Department of Biochemistry, Faculty of Science, King Abdulaziz University, Jeddah 21551, Saudi Arabia

**Keywords:** plant immunity, chaperone, ROS, phytohormones, MAPKs

## Abstract

In eukaryotes, mitogen-activated protein kinase (MAPK) cascades are highly conserved signaling modules in transducing extracellular signals perceived by receptors/sensors. MAPKs phosphorylate cytoplasmic and nuclear target proteins, modifying their stability, protein–protein interaction, enzyme activity, DNA-binding affinity, and subcellular localization. A comparative phosphoproteomics analysis of wild-type and *mapk* mutant plants identified a previously uncharacterized protein, USP1, as a novel MAPK target. In this work, we investigated the role of USP1 in pathogen responses. CRISPR-Cas9-generated knockout *usp1* mutants displayed enhanced resistance to the bacterial pathogen *P. syringae pv. tomato DC3000*, reduced apoplast reactive oxygen species (ROS) accumulation, and upregulated defense marker genes such as WRKY29 and FRK1. Transcriptome analyses revealed that immune hormone signaling genes, such as salicylic acid (SA) and jasmonic acid (JA), are differentially regulated. These hormones are responsible for primary defense responses against biotrophic and necrotrophic pathogens, respectively. Accordingly, *usp1* mutants show enhanced and reduced resistance to bacterial and fungal infection by *Pst* DC3000 and *Botrytis cinerea*, respectively. Biochemical analyses showed that USP1 exhibits chaperone-like activity that stabilizes various proteins, including the MAPKs MPK4 and MPK6. Furthermore, *usp1* mutants were defective in flg22-mediated immune priming, indicating that USP1 is required for the establishment of PAMP-induced immune memory. Collectively, our findings identify USP1 as a MAPK-regulated chaperone that plays an important role in coordinating plant immune responses.

## 1. Introduction

Plants are sessile organisms that are constantly exposed to numerous microbial pathogens; however, for a given plant host, only a few types of pathogens can cause disease. Unlike mammals, plants lack mobile defender cells and a somatic adaptive immune system, and instead rely on their innate immune system to counteract pathogens during infection [1]. During the course of evolution, plants have developed a defense system that effectively controls infections caused by microbial pathogens. Pathogenic bacteria enter plant tissues through natural openings such as stomata, hydathodes, or wound sites, and subsequently proliferate in the apoplast [2].

Plants have a multilayered defense system, in which the first layer of plant defense is provided by preformed physical and chemical barriers, including the cuticle, the cell wall, and secondary metabolites such as glucosinolates, camalexin, and phytoalexins [3]. The second, inducible layer of defense is initiated when plasma membrane-localized pattern recognition receptors (PRRs) detect conserved microbe-derived molecules known as microbe- or pathogen-associated molecular patterns (MAMPs or PAMPs) [4]. A well-studied example is the perception of bacterial flagellin by the receptor kinase FLS2, which restricts bacterial infection [5,6]. Elicitors generated as a result of host damage are referred to as damage-associated molecular patterns (DAMPs) [4]. The collective defense responses activated by PAMPs are termed PAMP-Triggered Immunity (PTI) [2,7]. To overcome PTI, adapted bacterial pathogens have evolved effector proteins that are secreted directly into host cells via the type-III secretion system (T3SS), suppressing PTI and resulting in Effector-Triggered Susceptibility (ETS) [8]. In response, plants have evolved intracellular resistance (R) proteins that recognize specific pathogen effectors, activating a robust defense response known as Effector-Triggered Immunity (ETI) [1,2]. This ongoing co-evolutionary arms race between plants and pathogens has shaped a highly dynamic and layered immune system.

Mitogen-activated protein kinase (MAPK) cascades are evolutionarily conserved signaling modules that regulate a broad range of biological responses to external stimuli, including pathogen attack, into intracellular responses [9]. In *Arabidopsis thaliana* (hereafter, *Arabidopsis*), nearly 110 genes encode MAPK cascade components, including 20 MAPKs, 10 MAPKKs, and 80 MAPKKKs [10,11], of which, MKK1 and MKK2 act upstream of MPK4 and are required for plant immunity [12,13] and MPK4 negatively regulates salicylic acid-dependent systemic acquired resistance [14], while MKK4, and MKK5 activate MPK3 and MPK6, which are central positive regulators of PTI [15]. Despite the complexity of MAPK signaling networks, only a fraction of MAPK substrates has been identified to date. Using flg22 as a MAMP, a comparative phosphoproteomic study conducted in wild-type and *mapk* mutant plants identified 152 differentially phosphorylated peptides, of which 70 were classified as putative MAPK targets involved in chromatin remodeling, transcriptional regulation, and chromatin-associated processes [16]. Among these 70 targets, At1g11360 (USP1) was selected for further characterization. Emerging evidence indicates significant crosstalk between biotic and abiotic stress response pathways, suggesting USP1 may play a dual role.

USP1 (At1g11360) is a 26.5 kDa protein belonging to the Universal Stress Protein (USP) family, characterized by a conserved UspA domain of 140–160 amino acids (Pfam Accession Number PF00582). The USP family was first described in bacteria, where UspA synthesis in *Escherichia coli* was shown to be induced by a broad spectrum of environmental stresses [17]. USP family members are widely distributed across most living organisms, including bacteria, archaea, fungi, protozoa, plants, and mammals, collectively encoding more than 1000 distinct proteins [18]. USP genes encode either a small protein of approximately 15 kDa harboring a single UspA domain, or a larger protein of approximately 30 kDa consisting of two tandem UspA domains. The UspA domain adopts an alpha/beta subdomain structure that is vital for stress-resistant metabolic and cellular defense pathways [19,20,21]. *Arabidopsis* contains 44 USP domain-containing proteins related to the *Methanococcus jannaschii* USP family [22], which are classified into two categories based on the presence or absence of an ATP-binding motif at their C-terminal region [23]. *Arabidopsis* USP proteins fall within the ATP-binding motif category (Figure S1).

Although USP genes have been identified in diverse plant species, including *Hordeum vulgare*, *Oryza sativa*, *Gossypium arboreum*, *Astragalus sinicus*, *Vicia faba*, and *Solanum pennellii*, their functions remain largely unknown [24,25,26,27]. Notably, two USP-like proteins in *Arabidopsis*, AtPHOS32 (At5g54430) and AtPHOS34 (At4g27320), have been identified as MAPK targets that are phosphorylated following flg22 treatment, with a conserved serine phosphorylation site located in the N-terminal region of the protein [28]. This evolutionarily conserved phosphoserine is absent in bacterial UspA proteins, and the functional significance of this phosphorylation event remains to be determined (Figure S2). USPs are known targets of serine/threonine phosphorylation in plant signaling cascades such as the MAPK pathway, and USP domains can act as modular scaffolds that facilitate stress adaptation and protein–protein interactions [22,28]. Several reports indicate that USP-like proteins play important roles in transport and scaffolding [29], and the distribution of USP domains across diverse species underscores their importance in plant growth, development, and stress responses.

There are numerous examples of USP proteins functioning as molecular chaperones and redox proteins [30,31,32]. AtUSP (At3g53990), a paralog of USP1, exhibits chaperone activity and is significantly induced by oxidative stress and H_2_O_2_ treatment [33]. The role of AtUSP is mechanistically parallel to that of the pathogen-responsive regulator NPR1 (Non-expressor of Pathogenesis-Related genes 1 [34]), whose conformation is regulated by the redox protein thioredoxin (Trx), triggering a shift from an oligomeric to monomeric form that enables nuclear translocation and activation of defense genes [35,36]. Similarly, cysteine residues in USP proteins have been shown to play a crucial role in regulating their redox state, suggesting that USPs may participate in redox-dependent immune signaling [31]. Despite these molecular properties, there are comparatively few reports on the role of USPs in plant defense against pathogens, and the contribution of USP1 to biotic stress responses has not been previously investigated.

While Manickam et al. [37] recently demonstrated USP1’s role in abiotic stress thermopriming, the present study investigates whether USP1 functions in biotic stress immunity via distinct molecular mechanisms, using physiological, transcriptomic, and biochemical analyses. We generated *usp1* loss-of-function mutants using CRISPR-Cas9 technology and demonstrated that *Arabidopsis usp1-1* and *usp1-2* knockout lines exhibit elevated basal immune levels and enhanced resistance against bacterial pathogens, implicating USP1 as a novel regulator of the plant immune response. Global transcriptome profiling of mutant lines following flg22 challenge was carried out to identify differentially expressed immune-related genes. Furthermore, using differential scanning fluorimetry (DSF), we show that USP1 functions as a molecular chaperone, protecting substrate proteins, including MPK4 and LDH, from thermal denaturation and maintaining their native conformation. We also demonstrate that *usp1* lines are defective in flg22-mediated immune priming, linking USP1 chaperone function to the establishment of immune memory. Collectively, this study provides mechanistic evidence that USP1 functions as a molecular chaperone that regulates plant immunity.

## 2. Results

### 2.1. USP1 Negatively Regulates MAMP-Triggered Innate Immunity

**Figure 1:**
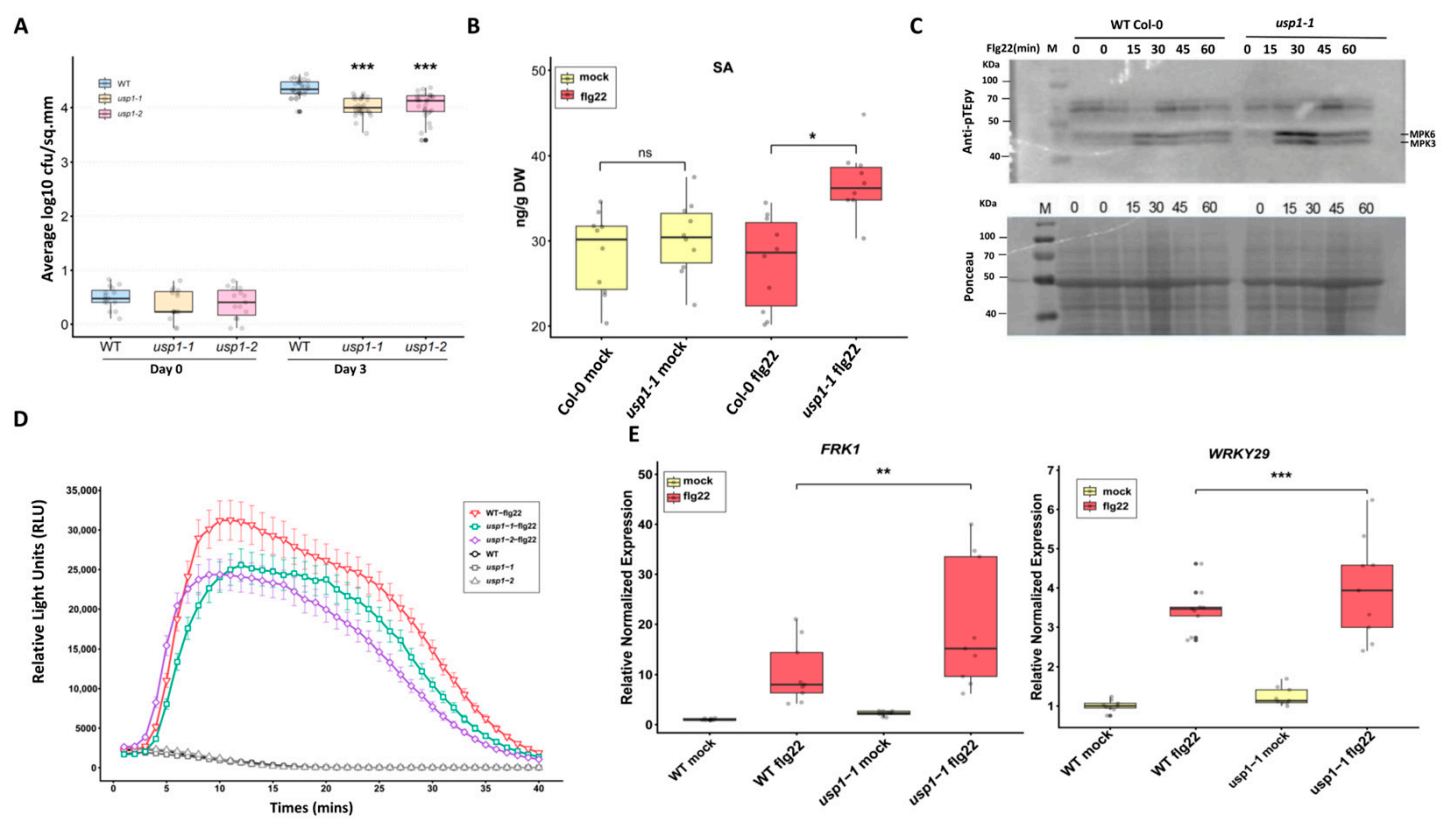
USP1 negatively regulates MAMP-triggered innate immunity. (**A**) Bacterial growth in *Arabidopsis* leaves inoculated with *Pseudomonas syringae* pv. *tomato* DC3000 in wild-type Col-0, *usp1-1*, and *usp1-2* plants. Four-week-old plants were spray-inoculated with a bacterial suspension or mock inoculation. Bacterial colonies were quantified at 3 and 72 hpi. Vertical bars indicate the standard deviation for the three biological replicates with 16 plants per treatment. Lines in the boxes indicate the median. (**B**) Quantitative analysis of SA in Col-0, *usp1-1*, and *usp1-2* lines analyzed by LC MS/MS, with or without flg22 treatment for 1 h. Samples of 100 mg/tube were taken from eighteen plants in three biological replicates of each line. (**C**) Flg22 rapidly and transiently activates MPK3 and MPK6 in mock and *usp1-1* mutants. The plants from WT (Col-0) and *usp1-1* mutants were incubated overnight in water and then treated for the indicated time with 1 µM flg22. MPK3 and MPK6 phosphorylation was monitored by Western blot using an anti-pTE-pY antibody against the phosphorylated form of ERK2. Coomassie staining shows the equal loading of the protein. (**D**) Flg22-induced ROS production in *usp1* mutants. Leaf disks were obtained from four-week-old *Arabidopsis thaliana*, WT Col-0, *usp1-1*, and *usp1-2*, and then treated with (1 µM flg22) or mock treatment. ROS accumulation, represented as Relative Light Units (RLUs), was detected by luminol-based assay over 40 min. Three independent experiments were carried out with similar results. (*n* = 12/treatment). (**E**) The relative expression levels of PTI marker genes in WT (Col-0), *usp1-1*, and *usp1-2.* The values were normalized to two reference genes *UBQ (At4g05050)* and *ACTIN2 (At3g18780).* The X-axis shows the expression levels of *FRK1* and *WRKY29* in *Arabidopsis* lines under mock and flg22 treatment. Y-axis shows the differences in levels of gene expression plotted using a log2 scale. The error bars represent SEM, the standard error mean (one asterisk (*) indicates *p* < 0.05, (**) *p* < 0.01, (***) *p* < 0.001; Student’s *t* test). Three independent replicates (*n* = 9) were performed for each experiment.

To investigate the role of USP1 in plant defense, the two CRISPR-Cas9-generated *usp1* (*usp1-1* and *usp1-2*) mutant lines and wild-type (Col-0) plants were challenged with the hemibiotrophic bacterial pathogen *P. syringae pv. tomato DC3000.* At 3 h post-inoculation (hpi), bacterial growth in the mutants was comparable to that in wild-type plants. However, by 72 hpi, bacterial titers were significantly reduced in both *usp1* mutant lines, suggesting that USP1 functions as a negative regulator of defense responses against *P. syringae* (Figure 1A). To determine whether altered immunity is associated with changes in defense hormone accumulation, we measured levels of the key hormone regulating plant immunity, salicylic acid (SA), following flg22 treatment. The data show a significant increase in SA accumulation in *usp1-1* mutant compared to WT after flg22 treatment and a change in the mock condition, although the difference is insignificant (Figure 1B). The activation of MPK3, MPK4, and MPK6 in response to flg22 was also affected: *usp1-1* mutant showed higher MPK3 and MPK6 activation than wild-type after 15 and 30 min of flg22 elicitation in seedlings (Figure 1C).

We also assessed early PAMP-triggered immune (PTI) responses to flg22 using ROS burst, MAPK activation, and PTI marker gene expression. Compared with WT plants, *usp1-1* mutant plants produced significantly less ROS following treatment with bacterial PAMP, flg22 (Figure 1D).

To further evaluate PTI signaling, the expression of two early PTI marker genes, *FRK1* and *WRKY29*, was examined in wild-type and *usp1-1* mutant plants under mock treatment or flg22 treatment. No expression was observed in the WT and mutants under mock conditions. After flg22 treatment, the *FRK-1* and WRKY29 transcript levels were significantly higher in the mutant compared to the wild-type (Figure 1E). Collectively, these results indicate that USP1 negatively regulates the PTI signaling.

### 2.2. USP1 Controls the Transcription of Defense Genes

**Figure 2:**
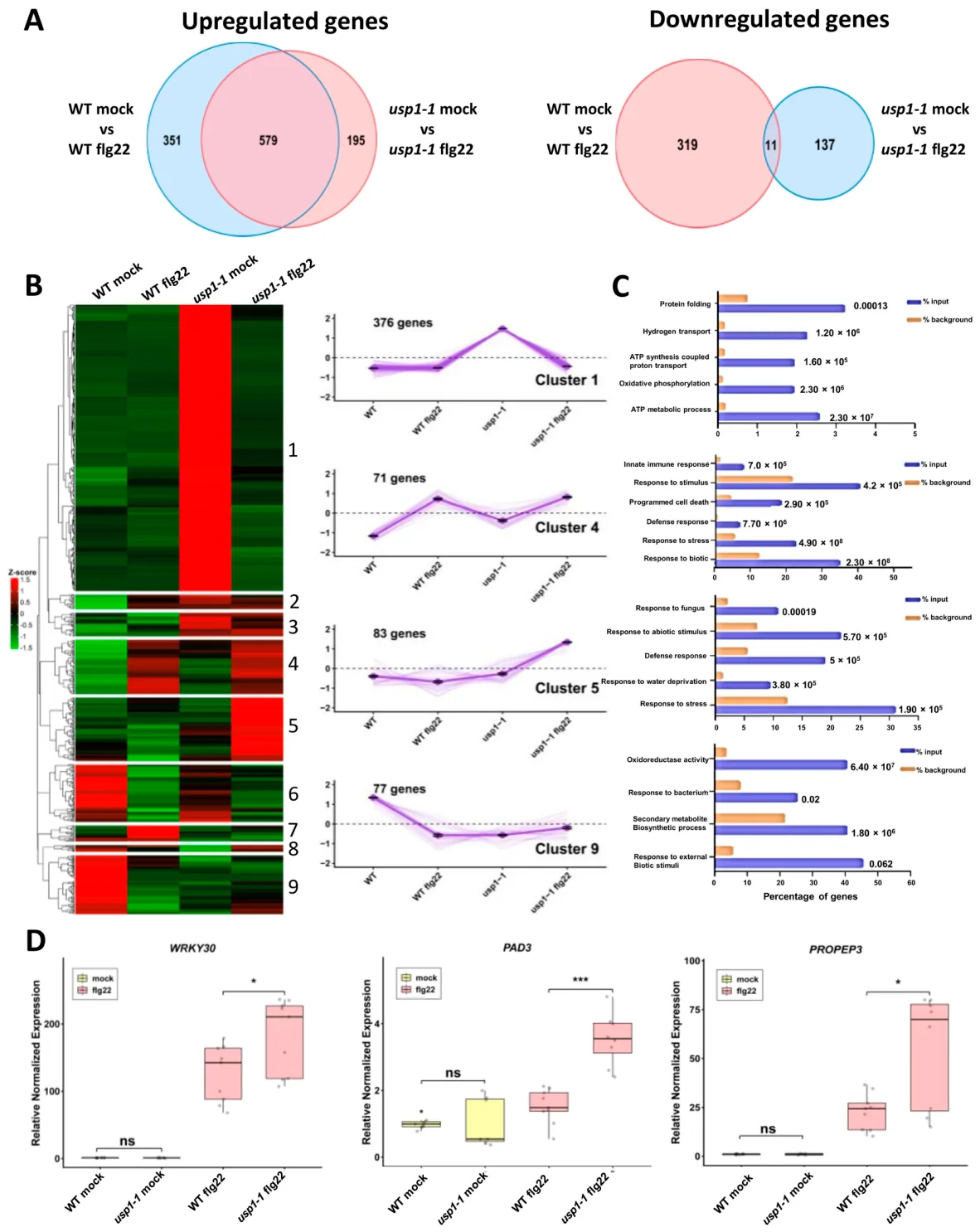
Characteristics of DEGs in *Arabidopsis usp1-1.* (**A**) Venn diagram depicting the overlap of differentially expressed genes (DEGs) among WT (Col-0) mock vs. WT-flg22, and *usp1-1* mock vs. *usp1-1* flg22 identified by RNA-Seq. (**B**) Hierarchical clustering graph of the 1592 DEGs in WT mock, WT-flg22, *usp1-1* mock and *usp1-1* flg22. The values are expressed as log 2-fold changes (*p* < 0.01). The average linkage method and Pearson correlation (MEV4.0) were used in the clustering. Red and green indicate upregulated and downregulated expression, respectively. The DEGs were classified into nine clusters. Black lines show the average values for relative expression levels, and gray lines represent the relative expression of each gene in clusters 3, 5, 8, and 9. (**C**) Functional Enrichment of DEGs between *Arabidopsis* Col-0 (WT) mock, *usp1-1* mock, WT flg22, and *usp1-1* flg22 in the GO databases. Blue represents input, and orange represents the reference control. (**D**) Validation of RNA-seq expression data by (RT-qPCR). The x-axis shows the expression of genes in WT mock, WT flg22-treated, *usp1-2* mock, and *usp1-2* flg22-treated plants in the *Arabidopsis* Col-0 background. The y-axis shows the differences in levels of gene expression, which were plotted using a log2 scale. Relative gene expression was calculated using the 2^(−ΔΔCt) method with *UBQ (At4g05050)* and *ACTIN2 (At3g18780)* as internal reference genes. Data represent mean ± SEM from three independent biological replicates, each with three technical replicates (*n* = 9 per condition). The error bars represent SEM, the standard error mean; ns indicates non-significant, one asterisk (*) *p* < 0.05and (***) *p* < 0.001; Student’s *t* test).

To gain deeper insight into the role of USP1 in plant defense, comparative RNA-seq transcriptome analysis was performed using WT (Col-0) and *usp1-1* mutant *Arabidopsis* plants treated with mock or flg22. The Venn diagram in Figure 2A summarizes differentially expressed genes (DEGs), both up- and downregulated genes, identified across four conditions: WT-mock, WT-flg22, *usp1-1*-mock, and *usp1-1*-flg22, as well as the number of overlapping DEGs between the four sets. In total, 1592 DEGs were identified, comprising 1125 upregulated and 467 downregulated genes. Hierarchical clustering grouped the DEGs into nine clusters, based on similar expression patterns (Figure 2B). Among all clusters, clusters 1, 4, 5, and 9 displayed distinct transcriptional profiles. For example, cluster 9 shows downregulation of genes in the *usp1-1* mutant compared to WT in the mock condition and no remarkable differences in flg22 treatment, while cluster 4 shows the opposite pattern to cluster 9. On the other hand, cluster 5 shows an upregulation of genes in the *usp1-1* mutant after flg22 treatment. Cluster 1 displays a significant upregulation of genes in *the usp1-1* mutant in the mock condition (Figure 2C). Interestingly, gene ontology (GO) analysis revealed that defense response-related terms were predominantly enriched in clusters 4, 5, and 9, while protein folding, oxidative phosphorylation, and ATP processes were enriched in cluster 9. Finally, the expression levels of three defense genes were validated by quantitative real-time PCR (qRT-PCR). Consistent with the transcriptome analysis, the transcript levels of *PAD3*, *PROPEP3*, and *WRKY30* (cluster 5) were significantly higher in the mutant than in the WT plants in response to flg22 treatment (Figure 2D).

### 2.3. USP1 Mutation Alters Hormone Pathways

**Figure 3:**
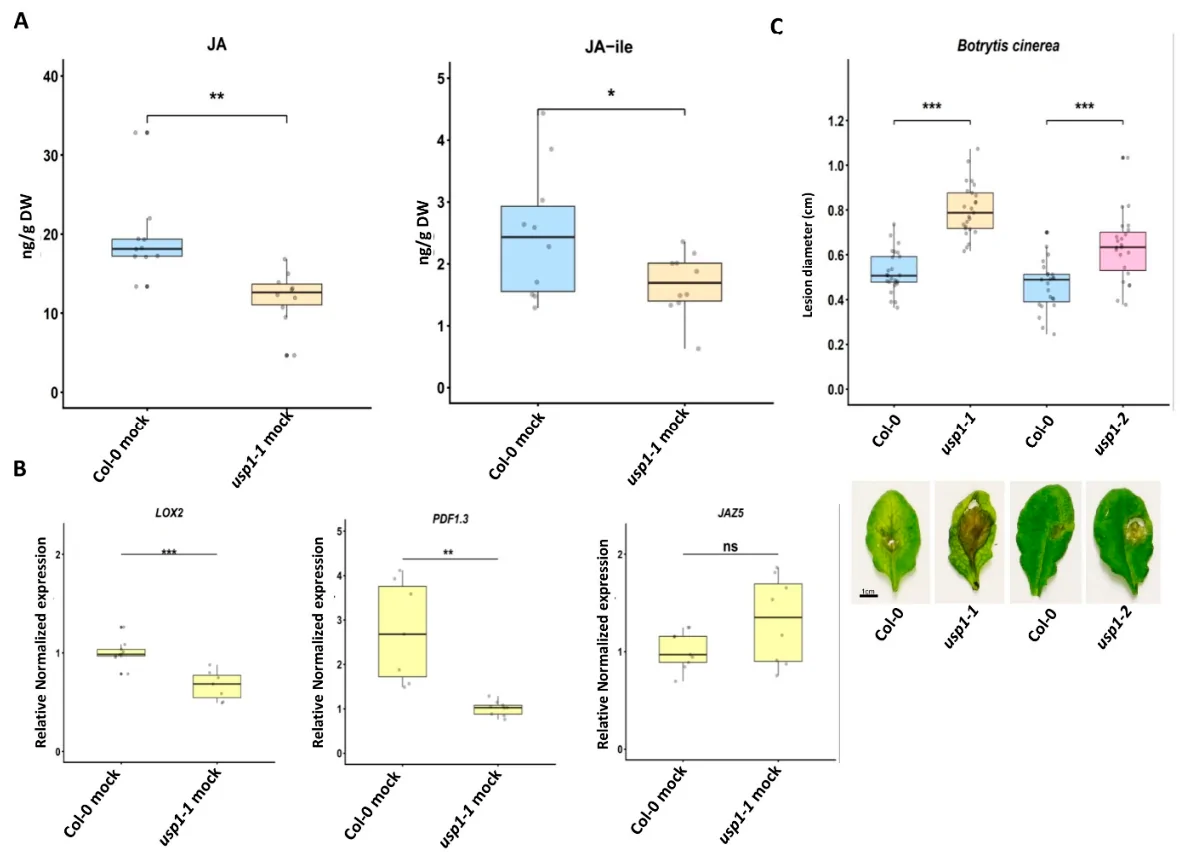
USP1 mutation alters hormone pathway and fungal resistance. (**A**) Quantitative analysis of JA and JA-lle levels in Col-0 (WT), *usp1-1*, *usp1-2* lines analyzed by LC MS/MS, with or without flg22 treatment for 1 h. Samples of 100 mg/tube were taken from eighteen plantsd in three biological replicates of each line. (**B**) qRT-PCR validation of *JAZ5* and *PDF1.3* expression in wild-type and *usp1-1* plants under mock and flg22 conditions. Expression was normalized to *ACTIN* and *UBQ10*. (**C**) The *Arabidopsis usp1* mutations enhanced susceptibility to the fungal *B. cinerea.* Four-week-old *A. thaliana* plants were inoculated with 5 μL droplets of fungal spores (5 × 10^5^ spores mL^−1^). The lesion area on the leaves of Col-0 and *usp1* mutant plants was measured at 72 hpi. Lesion sizes were measured using ImageJ software v1.53r. Vertical bars indicate the standard error for three biological independent experiments (*n* = 8). For each experiment, three leaves per plant of eight plants per replicate were used. The error bars represent SEM, the standard error mean; ns indicates non-significant, one asterisk (*) *p* < 0.05, two (**) *p* < 0.01 and (***) *p* < 0.001; Student’s *t* test). Representative infected leaves are shown below the graph.

Transcriptome analysis identified several DEGs that encode components of pathways related to phytohormone synthesis or signaling pathways. To further investigate the role of USP1 in hormone-mediated immunity, we quantified the levels of two key defense hormones, salicylic acid (SA; Figure 1B) and jasmonic acid (JA). The data show a significant decrease in JA, and its bioactive conjugate JA-Ile was reduced in the mutant compared to WT under the mock condition (Figure 3A).

Consistent with the hormone measurements, qRT-PCR analysis showed that JAZ5, which encodes a repressor of JA signaling, was altered in the mutant in response to flg22. Similarly, the transcript level of plant defensin (*PDF 1.3*) that is known for its antifungal activity was altered in the mutant in mock and flg22 treatment (Figure 3B). These data suggest that USP1 is involved in the regulation of JA-mediated defense responses. Given the reduced JA levels and altered expression of JA-responsive genes, we hypothesized that *usp1* mutants would exhibit compromised resistance to necrotrophic pathogens. To test this, we challenged four-week-old wild-type Col-0, *usp1-1*, and *usp1-2* plants with necrotrophic fungus *Botrytis cinerea*, and we observed that both *usp1-1* and *usp1-2* mutants displayed significantly larger lesions compared to the wild-type, indicating enhanced susceptibility (Figure 3C). To further investigate the molecular basis of this susceptibility, we analyzed the *Botrytis* defense pathway and defense-related genes, including analysis of *LOX2* expression, camalexin levels, and ABA content in the mutant (Figure 3B and Figure S3). These data demonstrated that USP1 is required for effective defense against necrotrophic fungal pathogens, likely through its role in maintaining proper JA-dependent defense signaling.

### 2.4. USP1 Shows Chaperone Activity in the LDH, MPK4, and MPK6

To investigate the biochemical function of USP1, we performed differential scanning fluorimetry (DSF), which monitors protein thermal stability and ligand-induced stabilization. Purified recombinant USP1 protein was tested together with Lactate Dehydrogenase (LDH), used as a model substrate (described in [37]), and the MAPKs MPK4 and MPK6 to assess potential protein-stabilizing activity in vitro. Experimental conditions, including SYPRO dye concentration and protein concentration, were optimized prior to analysis. Based on these results, DSF assays were performed in phosphate buffer, 1× concentration of SYPRO, and 0.5 μM of proteins. By using thermal destabilization, the DSF test has been widely utilized to deduce LDH interaction with substrate proteins in thermal stability tests.

**Figure 4:**
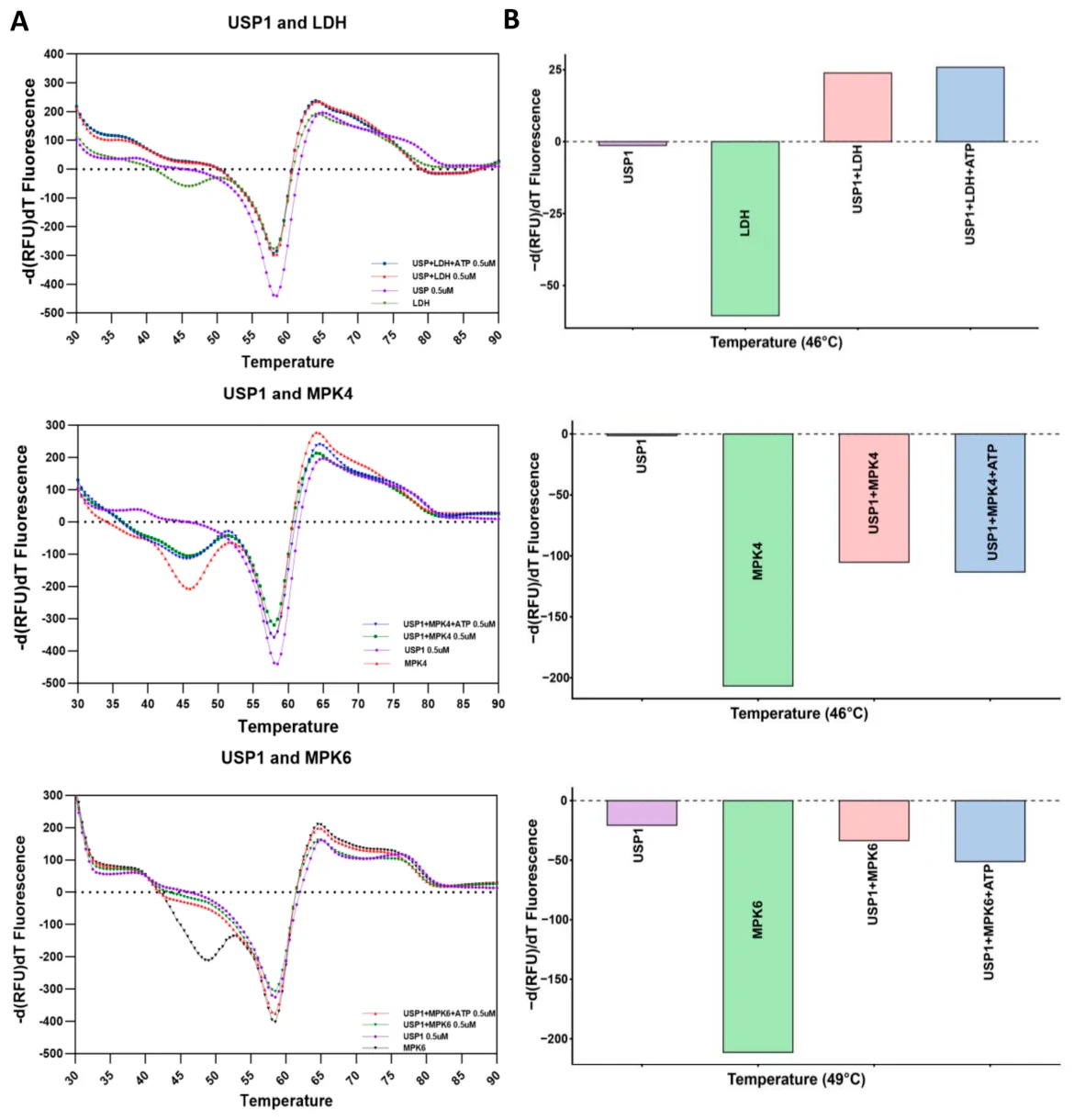
USP1 exhibits molecular chaperone activity. (**A**) Melting curve shows protein USP1, LDH, MPK4, and MPK6 unfolding by increase in temperature, accompanied by an increase in SYPRO fluorescence. The melting temperature (Tm) values were determined by calculating the first derivative from the melting curve using the Biorad CFX Manager software v3.1. (**B**) The X-axis temperature value that corresponds to the Y-axis value (−d(RFU)/dT) in the resulting curve resembles Tm.

In the absence of USP1, LDH exhibited thermal destabilization at 45 °C, whereas the addition of USP1 increased LDH thermal stability, suggesting a chaperone-like stabilizing effect. Similarly, MPK4 displayed an apparent melting temperature (Tm, app) of approximately 46 °C in the absence of USP1 (Figure 4A,B). Addition of USP1 resulted in a positive shift of 0.5 °C in the MPK4 melting curve (MPK4 Tm, app = 46 ± 0.5 °C), and destabilization of MPK4 alone at 46 °C, whereas the USP1 treatment maintained the native structure, indicating that USP1 interaction stabilized the MPK4 protein. Similarly, MPK6 had a melting point (Tm, app) of 49 °C in the absence of USP1. The addition of USP1 protein caused a positive shift of 0.5 °C in the melting curves (MPK6 Tm, app = 49 ± 0.5 °C), indicating that USP1 binds and stabilizes MPK6 (Figure 4A,B). The ability of USP1 to stabilize MPK4 and MPK6 is particularly relevant, as the MAPK signaling cascades regulate plant immunity.

### 2.5. Structural Modeling of USP1-MPK4/MPK6 Interaction

**Figure 5:**
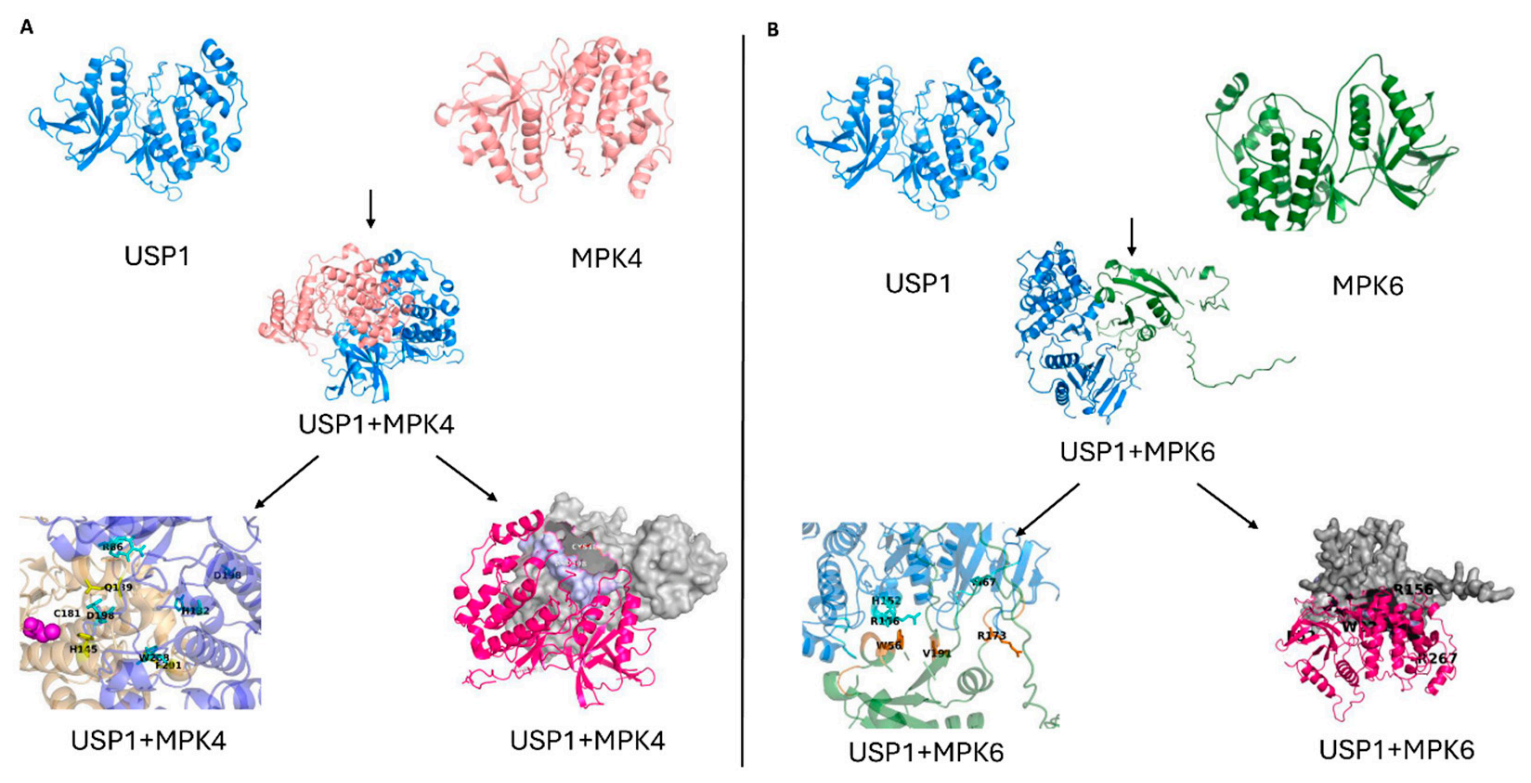
Protein–protein docking analysis of USP1 with MPK4 and MPK6. Left: (**A**) USP1 (blue) docked with MPK4 (salmon, PDB: 7W5C) using ClusPro (ΔG = −8.0 kcal/mol, Kd = 1.4 μM). Interface close-up shows key residues (USP1: R86, H132, D198, W208; MPK4: Q139, H145, C181, F291). CYS181 of the CD domain (magenta) is at the interface. Surface view shows MPK4 (grey) with USP1 (magenta) and binding pocket (light blue). (**B**) USP1 (blue) docked with MPK6 (green, PDB: 5CI6) using HDOCK. Key residues shown (USP1: R156, W228; MPK6: K171, Q59, R63). Conserved SP sites (S12, S24) are indicated as spheres. Surface view shows MPK6 (grey) with USP1 (magenta).

To further investigate the structural basis of the USP1-MPK4/MPK6 interaction, we performed protein–protein docking analysis. Docking of USP1 with MPK4 revealed a stable complex with a predicted binding affinity of −8.0 kcal/mol and a dissociation constant (Kd) of 1.4 μM, comprising 44 intermolecular contacts predominantly driven by hydrophobic interactions (17 apolar–apolar and 13 apolar–polar contacts). Notably, CYS181 of the MPK4 common docking (CD) domain was found at the binding interface, confirming that USP1 interacts through the canonical MAPK substrate recognition site (Figure 5). Similarly, docking of USP1 with MPK6 identified key contact residues, including R156 and W228 of USP1 and K171 and Q59 of MPK6. Multiple sequence alignment of USP homologs revealed a highly conserved SP (Ser-Pro) phosphorylation motif from archaea (*M. jannaschii*) through dicots (*Arabidopsis*, *Gossypium*) to monocots (*Oryza*, *Hordeum*, *Zea mays*), representing the canonical MAPK phosphorylation consensus sequence (Figure S2).

### 2.6. USP1 Is Required for flg22-Mediated Immune Priming

We previously demonstrated that USP1 functions as a molecular chaperone and is required for thermopriming in *Arabidopsis* [37]. Given that USP1 stabilizes MPK4 and MPK6, we hypothesized that USP1 may also be required for flg22-mediated immune priming, a process through which prior exposure to PAMPs enhances subsequent pathogen resistance. To test this hypothesis, we pre-treated wild-type, *usp1-1*, and *usp1-2* plants with flg22 (priming treatment) and subsequently challenged them with *Pseudomonas syringae* pv. *tomato* DC3000. Bacterial populations were quantified at two time points, 3 h post-inoculation (3 hpi) and 72 hpi, and two conditions, pathogen assay without priming (PA) and flg22 priming followed by pathogen assay (F + PA).

At 3 hpi, all three genotypes showed similar basal bacterial levels. At 72 hpi, in the unprimed pathogen assay (PA), the bacterial population was significantly higher in wild-type plants compared to both *usp1-1* and *usp1-2* mutants (Figure 5A), consistent with our earlier observation that *usp1* mutants exhibit enhanced basal resistance to *Pst* DC3000 (Figure 1A).

**Figure 6:**
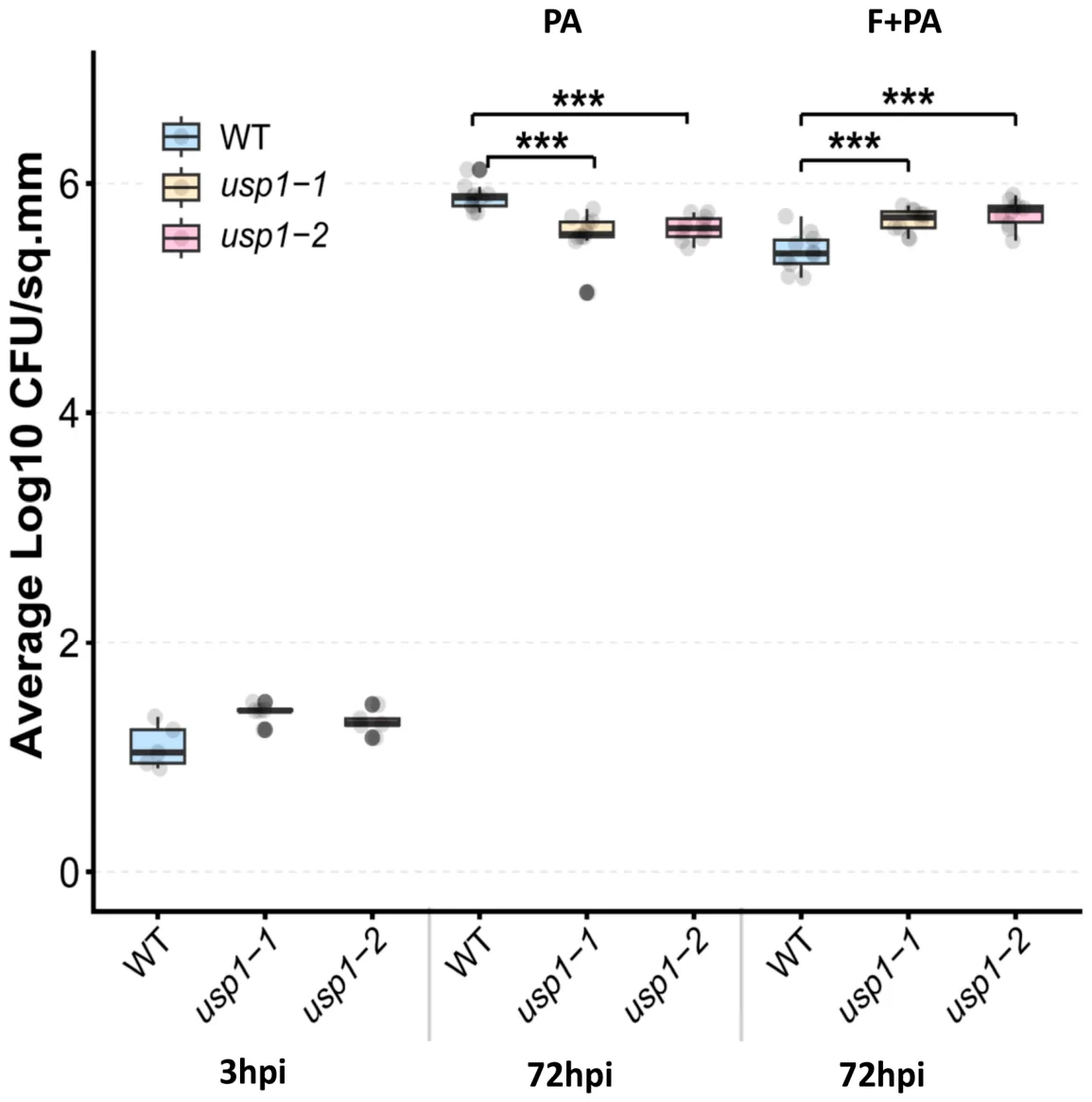
USP1 is required for flg22-mediated immune priming. Bacterial growth in *Arabidopsis* leaves of wild-type Col-0, *usp1-1*, and *usp1-2* plants under two conditions: pathogen assay without priming (PA), and flg22 priming followed by pathogen assay (F + PA). Four-week-old plants were pre-treated with 1 µM flg22 or water 24 h prior to spray-inoculation with *Pseudomonas syringae* pv. *tomato* DC3000 (10^8^ CFU/mL). Bacterial colonies were quantified at 3 and 72 hpi and expressed as Log_10_ CFU/sq.mm. In the PA condition, *usp1* mutants exhibit significantly lower bacterial levels than wild-type, confirming enhanced basal resistance. In the F + PA condition, wild-type plants show reduced bacterial levels compared to the PA condition, demonstrating effective immune priming, whereas *usp1-1* and *usp1-2* mutants show significantly higher bacterial levels than primed wild-type plants, indicating a defective priming response. Three independent biological replicates were performed with 16 plants per treatment. Error bars represent SEM (*** *p* < 0.001, Student’s *t* test).

Notably, under flg22-primed conditions (F + PA), wild-type plants pre-treated with flg22 showed a marked reduction in bacterial load compared to the unprimed PA condition, demonstrating a robust priming response. In contrast, both *usp1-1* and *usp1-2* mutants failed to mount an effective defense following flg22 priming, as their bacterial levels remained comparable to the unprimed condition and were significantly higher than those of primed wild-type plants (Figure 6). These results indicate that although *usp1* mutants have elevated constitutive defense responses, they are unable to mount an effective priming-induced immune response. These findings suggest that USP1 is required for flg22-mediated immune priming. The chaperone activity of USP1 may be essential for stabilizing immune signaling proteins to maintain a primed state. In the absence of USP1, destabilization of key signaling components could compromise the plant’s immune memory, leading to impaired priming-induced resistance to PAMPs during subsequent pathogen challenges.

## 3. Discussion

Plant MAPKs are activated in response to many biotic and abiotic stresses [38]. However, our knowledge of the substrates of MAPKs is limited. Protein kinases are mainly involved in cellular signal transduction via phosphorylating proteins in the cell. MAPK-dependent phosphorylation of their substrates and subsequent functional characterization is essential for understanding plant responses to various stresses. In this study, we investigated the function of the putative MAPK target USP1 and demonstrated that it plays a role in plant immunity by acting as a negative regulator of bacterial defense.

The *Arabidopsis* genome encodes 44 USP proteins, and USP1 was identified as a phosphorylated target in response to flg22 treatment in a phosphoproteomics screen [16]. Although the amino acid sequence of USP1 shares only 24.1% identity with the *Methanococcus jannaschii* MJ0577 UspA protein, the central 150-amino acid UspA domain is structurally conserved, and the ATP-binding residues identified in the crystal structure of MJ0577 [20] are retained in USP1. These observations suggest that USP1 may be regulated by ATP. We observed that serine S24 at the N-terminus of USP1 is conserved in related proteins across various plant species, including monocots and dicots [28], and our previous phosphoproteomic data confirmed that USP1 is phosphorylated at S24 by MPK3, MPK4, and MPK6 [16]. Two other Arabidopsis USP proteins (ATPHOS32 and ATPHOS34) are phosphorylated at the same serine site by MPK3 and MPK6 in response to flg22, although the functional importance of this phosphorylation has not been identified [28]. Together, these findings suggest that MAPK-mediated phosphorylation of USP proteins may represent an evolutionarily conserved regulatory mechanism. However, the functional relevance of the ATP-binding site in bacterial USP proteins remains unclear. Further studies will therefore be required to determine the biological significance of USP1 phosphorylation and ATP binding.

USP is known to positively regulate the response to drought and cold stress; here, we report that USP1 negatively regulates immunity in *Arabidopsis*. Notably, our phenotypic studies of *usp1* mutants enhanced resistance against *Pst DC3000*. Pathogen-triggered immunity (PTI) functions through a dynamic network of common signaling to activate defense responses against invading pathogens [39]. Upregulation of PTI marker genes (*WRKY29* and *FRK1*) was enhanced in the *usp1* mutant, suggesting that AtUSP1 negatively regulates the PTI signaling network. The oxidative burst is one of the earliest responses observed in plants treated with a MAMP elicitor (flg22). NADPH oxidases are the main producers of apoplastic ROS; superoxide generated by NADPH oxidases is rapidly dismutated into H_2_O_2_ [40,41,42,43]. Our data indicate reduced accumulation of apoplastic ROS in *usp1* mutants in response to flg22. These results suggest that the enhanced resistance phenotype of *usp1* mutants to infection by *Pst DC3000* might play a role in the transcriptional regulation of defense-related genes, and USP1 is a negative regulator of PTI.

### 3.1. USP1 Modulates Hormone Homeostasis and Defense Gene Expression

The transcriptome analysis in this study revealed that hormone signaling genes were deregulated in *usp1* mutants in response to flg22. Phytohormones play critical roles in plant disease resistance, serving as key regulators of defense responses against biotrophic and necrotrophic pathogens. During pathogen infection, the levels of hormones are increased, thereby activating the expression of defense genes [44]. Genes encoding cytochrome P450 family members and UDP-glycosyl transferases were strongly upregulated in the WT mock-treated plants, whereas no significant changes were observed in the *usp1-1* mock-treated plants (Figure S3). The cytochrome P450 family plays a vital role in plant growth and development, as well as in defense responses. P450 family enzymes are involved in the biosynthesis of several secondary metabolites such as terpenoids, flavonoids, and glucosinolates. P450 enzymes also regulate plant hormone metabolism [45]. Recent studies have reported that CYP450 enzymes are involved in phytoalexin biosynthesis and in the regulation of JA for plant defense responses [46]. UDP-glycosyltransferases (UGTs), including UGT73B3 and UGT76B1, have been implicated in SA–JA crosstalk during pathogen defense [47,48]. The upregulation of SARD1, a regulator of ICS1 induction and SA synthesis, in the *usp1* mutant plants further supports a role for USP1 in modulating hormone homeostasis. Whether USP1 directly regulates UGT activity requires further investigation.

### 3.2. USP1 Is Required for JA-Mediated Defense Against Botrytis cinerea

In our study, defense response genes were specifically induced upon flg22 treatment in *usp1* mutant Arabidopsis. *WRKY30*, and some regulators of the JA signaling pathway, including jasmonate-ZIM-domain protein 5 (JAZ5), were deregulated. JAZ proteins repress JA-responsive transcription factors in the absence of JA-Ile. Upon perception of JA-Ile, JAZ proteins dissociate from their transcription factors to form a co-receptor complex with COI1 before they are degraded. Then, JAZs release transcription factors that activate JA-responsive genes [49,50,51,52]. Genes encoding plant defensin proteins PDF 1.2 and PDF 1.3, and JA biosynthetic enzyme *LOX2*, were downregulated in the *usp1* mutant plants. *Arabidopsis* contains 13 defensin genes, including three *PDF 1.2* genes (*PDF1.2a*, *PDF1.2b*, and *PDF1.2c*). PDF1.2a was highly responsive to JA but insensitive to SA [53]. Mutants that are impaired in either JA or ET signaling do not express PDF1.2a and display enhanced susceptibility to necrotrophic pathogens [54]. The downregulation of *PDF1.2*, *PDF1.3*, and *LOX2* in the *usp1* mutant plants suggests that USP1 could be a positive regulator in the JA-dependent defense response to necrotrophic pathogens, and we propose that the repression of SA-mediated PTI signaling by USP1 occurs through the transcriptional activation of negative regulators of PTI.

While Manickam et al. [37] demonstrated USP1’s role in abiotic stress thermopriming through HSFA2 stabilization, this work reveals a distinct immune-pathway-specific function through MAPK stabilization. USP1 functions as a substrate-specific chaperone, stabilizing transcription factors (HSFA2) in abiotic stress and kinases (MPK4/6) in biotic stress, thereby coordinating memory across distinct pathways with phenotypes and downstream targets.

Both the transcriptome data and the qRT-PCR results showed that *usp1* mutant plants significantly affect a large number of SA- and JA-related genes. Therefore, we measured endogenous SA, JA, and JA-Ile hormone levels in the USP1 knockout and WT lines. Under untreated (mock) conditions, we observed a considerable decrease in JA and JA-Ile levels in *usp1* mutant plants. In agreement with the transcriptome data, we expected JA hormone levels to be lower in mutant plants. Low levels of JA have been linked to increased susceptibility to necrotrophic infections. As a result, we tested *usp1* mutant plants and the WT line for sensitivity to *Botrytis cinerea*, a necrotrophic fungal pathogen. The *usp1* mutants were more susceptible to *B. cinerea*, when compared to wild-type plants. Therefore, we conclude that USP1 also plays a role in fungal pathogen response by regulating the JA-related genes.

Furthermore, we identified increased SA accumulation levels in *usp1* mutant plants under flg22 treatment when compared to wild-type plants (Figure 1). The elevated SA response upon MAMP elicitation, combined with the reduced JA levels, is consistent with the antagonistic role of SA and JA-meditated defense signaling [55]. This explains the opposing phenotypes—enhanced resistance to *Pst DC3000* but increased susceptibility to *B. cinerea*. The upregulation of SARD1, a key regulator of ICS1-dependent SA biosynthesis, in the *usp1-1* mutant further supports the role of USP1 in modulating SA-JA homeostasis. NPR1 (non-expressor of PR genes 1), which is a well-known central player in SA signaling [56], as well as NPR3 and NPR4 proteins, have recently been identified as SA receptors [57]. We found that SA levels increased in *usp1* mutant plants, which suggests that USP1 protein levels suppress the SA signaling defense response. Further work is required to investigate the effect of USP1 and NPR1 during plant pathogen infection.

### 3.3. USP1 Confers Conformational Stabilization on MPK4 and MPK6

Using differential scanning fluorimetry, we identified a function for the USP1 protein as a molecular chaperone that helps protect the structures of MPK4 and MPK6. Our previous experiments demonstrated that USP1, MPK4, and MPK6 bind in vivo via BiFC and Co-IP [16], and in vitro DSF experiments are consistent with chaperone-like functions. This response indicated that USP1 functions as a molecular chaperone. The ability of USP1 to stabilize MPK4 is particularly significant in the context of plant immunity. Consistent with the experimental observation, our protein–protein docking analysis shows that CYS181 of the MPK4 CD domain is positioned at the binding interface, providing structural evidence that USP1 is recognized through the canonical MAPK substrate recognition site, as with other known MAPK substrates. This cysteine undergoes sulfenylation under oxidative stress [58,59], suggesting a potential redox-dependent regulation of the USP1-MPK4 interaction that may link ROS signaling to MAPK pathway modulation. Furthermore, the conserved SP motif identified across USP homologs from archaea to monocots is positioned to be accessible for phosphorylation by MPK4, supporting USP1 as a direct MAPK substrate and suggesting an evolutionarily ancient regulatory mechanism (Figure S2). MPK4 functions upstream of WRKY33, which directly activates PAD3 and CYP71A13 genes essential for camalexin biosynthesis, and WRKY33 is co-regulated by MPK4/6 and CPK5/6 [60,61]. Loss of USP1 chaperone activity could therefore destabilize MPK4, impairing the MPK4–WRKY33–PAD3 signaling axis and reducing camalexin production, which is consistent with the enhanced susceptibility of *usp1* mutants to *B. cinerea* (Figure S5).

### 3.4. Kinase Conformational Stability and MAPK Substrate Phosphorylation Efficiency

Protein chaperones have been characterized against various stresses, but emerging evidence reveals their additional roles as signaling regulators that modulate the temporal dynamics of kinase activity [61,62]. Our finding that USP1 associates with and stabilizes MPK4/6 through chaperone-like mechanisms suggests a regulatory function in plant immunity beyond canonical protein folding roles. There was also a modest but reproducible thermal stabilization observed in our DSF experiments (~0.5 °C Tm increase for USP1-MPK4 and USP1-MPK6 complexes). Recent structural and biochemical studies demonstrate that small shifts in kinase thermal stability can profoundly impact substrate accessibility and catalytic efficiency [63,64]. Kinase conformational stability directly determines the accessibility of the activation loop and substrate-binding pocket, two regions critical for phosphotransfer. By maintaining MPK4/6 in a competent catalytic conformation, USP1 likely extends the period during which these kinases can phosphorylate their downstream targets, particularly the transcription factor WRKY33. This model is consistent with recent mechanistic insights into kinase regulation by molecular chaperones. HSP90, the kinase-associated chaperone, maintains the active conformation of kinases through ATP-dependent processes that stabilize the N- and C-lobes of the kinase domain in a catalytically competent state [61]. While USP1 operates through distinct mechanisms (ATP-independent, direct protein–protein interaction), the functional outcome is sustained kinase activity. Consistent with this interpretation, *usp1* mutants show reduced expression of WRKY33-target genes (PAD3, CYP71A13), leading to compromised camalexin biosynthesis.

### 3.5. USP1 Is Required for flg22-Mediated Immune Priming

An important finding of this study is that *usp1* mutants are defective in flg22-mediated immune priming. While unprimed *usp1* mutants exhibit enhanced basal resistance to *Pst DC3000*, they fail to mount an effective defense following flg22 priming. This observation parallels the thermopriming defect previously reported for *usp1* mutants [37] and suggests that USP1 plays a conserved role in both biotic and abiotic stress memory. Immune priming involves the establishment of a “primed” state in which signaling components are maintained in a poised conformation, enabling stronger, recurring activation upon subsequent pathogen challenge [65,66,67,68]. The chaperone-like activity of USP1 may be critical for maintaining the stability and conformation of key signaling proteins during the priming phase. In the absence of USP1, the destabilization of these proteins may prevent the establishment of an effective primed state. These findings collectively position USP1 as a molecular chaperone for immune priming, providing mechanistic insight into how plants integrate stress memory across different environmental challenges.

In summary, our findings reveal that USP1 plays a multifaceted role in plant immunity. As a MAPK target with chaperone-like activity, USP1 modulates the stability and function of key immune signaling components. The *usp1* mutant phenotype characterized by enhanced bacterial resistance coupled with increased fungal susceptibility reflects the antagonistic relationship between SA- and JA-mediated defense pathways. USP1 thus represents a novel regulator that contributes to the balance between biotrophic and necrotrophic pathogen defense in *Arabidopsis*. Future studies should address several key questions: (i) Does MAPK-dependent phosphorylation of USP1 at S24 regulate its chaperone activity, and do phosphomimetic (S24D) or phospho-dead (S24A) variants alter MPK4 stabilization? (ii) What is the precise role of ATP binding in USP1 function? (iii) Does USP1 interact with the NPR1-mediated SA signaling pathway? Addressing these questions will clarify the molecular mechanisms by which this conserved stress protein integrates immune signaling in plants.

## 4. Materials and Methods

### 4.1. Plant Materials and Growth Conditions

*Arabidopsis thaliana* ecotype Columbia-0 (Col-0) was used as the wild-type (WT), and *usp1-1* and *usp1-2* were used as mutants [37]. Seeds were surface-sterilized and subjected to stratification at 4 °C on growth medium for a minimum of two days. The growth medium consisted of half-strength Murashige and Skoog basal salts with minimal organics, supplemented with 0.05% MES hydrate and 0.5% agar (Sigma A4675, St. Louis, MO, USA), and adjusted to pH 5.7 using KOH. Seedlings were cultivated under long-day conditions (16 h light/8 h dark) at 23 °C during the day and 22 °C at night for the specified duration.

For plants grown in Jiffy pots within Percival growth chambers (Percival Scientific, Perry, IA, USA), seeds were first stratified in water at 4 °C for at least two days, then grown under short-day conditions (8 h light/16 h dark) at 23 °C day/22 °C night and 60% relative humidity for four weeks. Plants were watered twice weekly with distilled water and fertilized with complete nutrient solution on alternating weeks. Illumination was provided by LED grow lights at an intensity of 120–150 μmol m^−2^ s^−1^.

### 4.2. Quantitative Real-Time PCR of MAMP-Induced Defense Genes

Fourteen-day-old WT and mutant *Arabidopsis* seedlings (six to eight seedlings per replicate) grown on ½ MS agar were transferred into ½ MS liquid medium overnight prior to treatment. The whole seedlings were treated with 1 µM flg22 peptide (QRLSTGSRINSAKDDAAGLQIA; synthesized by GenScript, Piscataway, NJ, USA) or water (mock) for 1 h. Lyophilised flg22 (purity > [95%], HPLC) was dissolved in sterile water, stored in aliquots at −20 °C and diluted into the medium immediately before use. Following treatment, 100 mg samples were homogenized in liquid nitrogen, and total RNA was extracted using the NucleoSpin Plant RNA kit (Macherey-Nagel, Düren, Germany). For each sample, 1 µg of total RNA was reverse-transcribed into cDNA using the SuperScript III First-Strand Synthesis Supermix kit (Invitrogen, Vilnius, Lithuania). The resulting cDNA was used as a template for qRT-PCR, with primers for the PTI marker gene, SsoAdvanced Universal SYBR Green Supermix (Bio-Rad, Munich, Germany) and qPCR (CFX384, Bio-rad, Hercules, CA, USA). ACTIN (At3g18780) and UBQ10 (At4g05320) were used as internal reference genes to normalize expression levels across all samples using Bio-Rad CFX Manager software v3.0. Primers for qRT-PCR are listed in Table 1. All experiments were performed with three independent biological replicates, each comprising three technical replicates. Normalized gene expression levels were expressed relative to wild-type controls in each experiment.

### 4.3. Bacterial Pathogen Infection Assay

Four- to five-week-old *Arabidopsis* wild-type (Col-0) and mutant plants grown in Jiffy pots under short-day conditions (8 h light/16 h dark) at 23 °C and 60% relative humidity were used for all inoculation assays. *Pseudomonas syringae pv. tomato DC3000* CUCPB5112 (a kind gift from Prof. Jane Parker, Max Planck Institute for Plant Breeding Research, Cologne (Koln), Germany) was cultured on NYGA plates, resuspended in 10 mM MgCl_2_ solution in the presence of 0.04% (*v*/*v*) Silwet L-77, and adjusted to OD_600 nm_ = 0.2. Bacterial inoculation was performed by spray-inoculating rosette leaves with a suspension of 10^8^ CFU/mL prepared as described earlier, covered with a transparent plastic lid. Inoculated plants were maintained in closed trays incubated in Percival growth chambers (Percival Scientific, Perry, IA, USA). Disease symptoms were evaluated at 3 and 72 h post-infection (hpi). To quantify bacterial growth in plants, leaf discs (0.5 cm diameter) were harvested from three different leaves of five plants per genotype at 3 hpi, and from ten plants per genotype at 72 hpi, as technical replicates. Leaf discs were surface-washed and sterilized with 70% ethanol for 10 s and washed with sterile distilled H_2_O twice, and then the internalized bacteria were extracted in 500 μL of 10 mM MgCl_2_ containing 0.01% (*v*/*v*) Silwet L-77. After serial dilution, homogenates were plated on LB agar medium supplemented with rifampicin (50 mg/L) and incubated at 28 °C. Bacterial colonies were counted at 48 h, and infection levels were expressed as CFU/cm^2^ of leaf area. Data represent the means of three independent biological replicates. The infection assays were repeated a total of three times.

### 4.4. Luminol-Based Apoplastic ROS Burst Assay

Apoplastic reactive oxygen species (ROS) production was measured using the luminol-based assay as described previously [68]. Leaf discs (4 mm diameter) from four-week-old WT and mutant *Arabidopsis* plants were incubated, adaxial side up, in white 96-well plates (Thermo Fisher Scientific, Catalog no: 236108, Waltham, MA, USA) containing 150 µL sterile water overnight. The following day, water was removed and replaced with 100 µL reaction solution containing 50 µM luminol (Sigma-Aldrich, St. Louis, MO, USA) and 10 µg/mL horseradish peroxidase (Sigma-Aldrich). Wells were treated with a final concentration of 1 µM flg22 or water as a mock control. Luminescence was recorded at one-minute intervals for 40 min following MAMP addition using a TECAN Infinite 200 PRO microplate reader (Tecan, Mannedorf, Switzerland) with a signal integration time of 0.5 s. ROS production was expressed as mean Relative Light Units (RLUs). Each time point represents the mean value of eight leaf discs. The data shown are representative of three independent experiments with consistent behavior.

### 4.5. Immunoblot Analysis of MAPK Activation (pTEpY Assay)

Fourteen-day-old WT and *usp1* mutant *Arabidopsis* seedlings grown on ½ MS agar plates were transferred into ½ MS liquid medium overnight and treated with flg22 or water (mock) for 0, 15, 30, 60 min. Whole seedlings were frozen in liquid nitrogen and ground using a tissue lyser (Tissue lyser II, Qiagen, Hilden, Germany) and resuspended in 200 µL SDS sample buffer, heated at 85 °C for 10 min, and centrifuged at 18,000× *g* for 10 min at 4 °C. The supernatant was collected for analysis. Total proteins were resolved by 10% SDS-PAGE and electro-transferred onto PVDF membranes (Bio-Rad, Hercules, CA, USA). Membranes were blocked with 5% (*w*/*v*) BSA in TBST at room temperature for 1 h, then incubated overnight at 4 °C with anti-phospho-p44/42 MAPK antibody (diluted 1:75,000 in 2% (*w*/*v*) BSA/TBST; #4370, Cell Signaling Technology, Danvers, MA, USA). HRP-conjugated goat anti-rabbit IgG was used as the secondary antibody (Promega, Madison, WI, USA). HRP activity was detected using ECL Prime chemiluminescent detection reagent (RPN2232, GE Healthcare, Chicago, IL, USA) and imaged on a ChemiDoc MP imaging system (Bio-Rad). Total protein loading was verified by Coomassie Blue staining and Ponceau S staining (P7170, Sigma-Aldrich, St. Louis, MO, USA). Each immunoblot shown is representative of three independent biological replicates.

### 4.6. RNA Sequencing and Transcriptome Analysis

Three independent biological replicates of 14-day-old WT and *usp1* mutant *Arabidopsis* whole seedlings grown on ½ MS agar were transferred into ½ MS liquid medium overnight, then treated with 1 µM flg22 peptide (QRLSTGSRINSAKDDAAGLQIA; synthesized by GenScript Biotech Corporation, Piscataway, NJ, USA) or water (mock) for 1 h. RNA-seq libraries were prepared using the Illumina TruSeq Stranded mRNA Sample Preparation LS kit according to the manufacturer’s protocol and sequenced on a HiSeq 4000 platform (Illumina, San Diego, CA, USA) with 150 BP paired-end reads and a read length of 300 bp fragment length. Raw reads were quality-controlled using FastQC v0.11.5 (http://www.bioinformatics.babraham.ac.uk/projects/fastqc/ accessed on 12 May 2022) and quality-trimmed using Trimmomatic v0.39 with the following parameters: minimum length of 36 bp; mean Phred quality score > 30; removal of leading and trailing bases below a quality of 3; and sliding window of 4:15. Trimmed reads were aligned to the *Arabidopsis thaliana* TAIR10 reference genome using TopHat v2.1.1, and transcript abundance was estimated as fragments per kilobase of transcript per million mapped reads (FPKM). Differentially expressed genes (DEGs) were identified using Cufflinks/Cuffdiff v2.2.1 [69] with quartile normalization and the limma package 3.68.5 in R 4.5.3. Genes exhibiting a ≥2-fold change between flg22-treated and mock-treated samples with an adjusted *p*-value ≤ 0.05 were considered significantly differentially expressed (Figure S4). Hierarchical clustering of DEGs was performed using MeV v4.8.1 [70]. Gene Ontology (GO) classification was performed using AgriGo v2 [71] and DAVID Bioinformatics Resources (https://davidbioinformatics.nih.gov/ accessed on 12 July 2022) [72]. Enrichment in GO terms was calculated with an adjusted *p*-value threshold of 0.05 and a minimum of five mapping entries per GO category. Venn diagrams were generated using the Venny online tool (http://bioinfogp.cnb.csic.es/tools/venny/ accessed on 12 July 2022). Enrichment figures were produced using the R package ggplot2 v 4.0.3. RNA-Seq data are deposited at NCBI Gene Expression Omnibus (GEO) under accession number GSE348911.

### 4.7. Phytohormone Analysis

Two-week-old WT and *usp1* mutant *Arabidopsis* seedlings grown on ½ MS agar were transferred into ½ MS liquid medium overnight, then treated with 1 µM flg22 peptide or water (mock) for 1 h. Phytohormone extraction was carried out as previously described [73]. Quantification was performed by HPLC-ESI-SRM using a Thermo Fisher TSQ-Altis Triple Quadrupole Mass Spectrometer coupled to a Thermo Scientific Vanquish MD HPLC system (Thermo Fisher Scientific, Waltham, MA, USA, and Agilent, Santa Clara, CA, USA). Chromatographic separation was performed on a UPLC column (Agilent Eclipse Plus C18, RRHD, 1.8 µm, 2.1 × 50 mm). Compounds were eluted using water (A) and acetonitrile (B) as mobile phases at a flow rate of 0.6 mL/min in gradient elution mode as follows: 10% B for 0.5 min, 10–55% B at 4.5 min, 55–100% B at 4.7 min, and 100% B until 6.0 min. Column temperature was maintained at 55 °C.

### 4.8. Botrytis cinerea Infection Assay

Four-week-old *Arabidopsis* wild-type (Col-0) and mutant plants grown in Jiffy pots under short-day conditions (8 h light/16 h dark) at 23 °C and 60% relative humidity were used for inoculation assays. *Botrytis cinerea* isolate 2100 (CECT2100; Spanish Type Culture Collection, Universidad de Valencia, Valencia, Spain) was used, which was cultivated on potato dextrose agar (PDA) plates at 22 °C for 10 days. Spores were collected, washed, and frozen at −80 °C in 0.8% NaCl at a concentration of 10^7^ spores mL^−1^. For inoculation of *Arabidopsis* plants, the spores were diluted in Vogelbuffer (in 1 L: 15 g of Sucrose, 3 g of Na-citrate, 5 g of K_2_HPO_4_, 0.2 g of MgSO_4_·7H_2_O, 0.1 g of CaCl_2_·2H_2_O, and 2 g of NH_4_NO_3_) to 5 × 10^5^ spores mL^−1^. using a hemocytometer. Five microliters of a *B. cinerea* conidial suspension (5 × 10^5^ spores/mL) were deposited onto each rosette leaf. Inoculated plants were immediately covered with a humidity dome to maintain high relative humidity and transferred to the growth chamber. Lesion area was photographed with the canon EOS camera (Canon, Tokyo, Japan) and quantified at 72 h post-infection (hpi) using ImageJ software v 1.5x (National Institutes of Health, Bethesda, MD, USA). Three independent biological replicates were performed, with each replicate consisting of nine plants per genotype.

### 4.9. Differential Scanning Fluorimetry (DSF)

Purified recombinant proteins USP1, MPK4, and MPK6 were prepared at concentrations of 0.5 µM, 1 µM, 5 µM, 10 µM, and 15 µM and combined with SYPRO Orange fluorescent dye (Thermo Fisher Scientific, Ref. S6650, Lot 2008138) in phosphate buffer at a final dye concentration of 5× in a total reaction volume of 10 µL per well in a 96-well microtiter plate. DSF experiments were performed using a Bio-Rad CFX96 real-time qPCR system in the all-channel fluorescence mode (Bio-Rad, Hercules, CA, USA). Samples were heated from 25 °C to 100 °C at a ramp rate of 2 °C/min, and fluorescence was continuously monitored throughout the temperature gradient. The melting temperature (Tm) for each protein was determined from the first derivative of the fluorescence signal using Bio-Rad CFX Manager software [74]. All experiments were performed in technical triplicate.

### 4.10. Protein–Protein Docking Analysis

Protein–protein docking simulations were performed to predict interactions between USP1 and mitogen-activated protein kinases (MPK4 and MPK6) using crystal structures obtained from the Protein Data Bank (PDB): MPK4 (PDB ID: 7W5C) and MPK6 (PDB ID: 5CI6). The USP1 protein structure was predicted using AlphaFold2 [75]. USP1 was docked with MPK4 using the ClusPro web server [76] with default parameters. USP1 was docked with MPK6 using the HDOCK web server [77] with an energy-based hierarchical algorithm. Top-ranked models were selected based on clustering analysis and binding free energy (ΔG). Key residues at the USP1-MPK4 and USP1-MPK6 interfaces were identified using the Protein Interfaces, Surfaces and Assemblies (PISA) server [78]. Hydrogen bonding, salt bridges, and hydrophobic interactions were analyzed using PyMOL (The PyMOL Molecular Graphics System, Version 2.5; Schrödinger, LLC).

## Figures and Tables

**Table 1:** Primers used for qPCR.

Name	Sequence	Target Gene	Purpose
P0130-F	GCGTAAATACGGGCAGAAAC	AT4G23550	WRKY29
P0131-R	GGTTTGGGTTGGGAAGTTTT	AT4G23550	WRKY29
P0126-F	ACGGGCATAGTTCCACAAAG	AT2G19190	FRK1
P0127-R	CGTCAAAAGAACGACGATGA	AT2G19190	FRK1
P0252-F	CGCTGGACGATGGATTCAGTTGGAGA	WRKY30	P0252-F
P0253-R	TCGGTTCGAGGTTTTGTATCGGCATTG	WRKY30	P0253-R
P0260-F	AGCAAGAGAACGATGGAGATGCT	PAD3	P0260-F
P0261-R	TCCCTGCTACAAATATGTCCGAGA	PAD3	P0261-R
P0266-F	CAACGATGGAGAATCTCAGA	PROPEP3	P0266-F
P0267-R	CTAATTGTGTTTGCCTCCTTT	PROPEP3	P0267-R
P0268-F	AGAAGTTGTGCGAGAAGCCA	PDF 1.3	P0268-F
P0269-R	ACACACCATGAAGCACCAAGT	PDF 1.3	P0269-R
P0258-F	ATGTCGTCGAGCAA TGAAAA	JAZ5	P0258-F
P0259-R	CTATAGCCTTAGAT CGAGAT	JAZ5	P0259-R
P0234-F	GAGCGACGAGAAGACGAAAGA	CYP76G1	P0234-F
P0235-R	ATCCGTTCCAGCCGTAAACA	CYP76G1	P0235-R
P0262-F	CACCTGACGAAGAGTACATTGG	LOX2	P0262-F
P0263-R	GTAACACCATGCTCAGAGGTAGG	LOX2	P0263-R
P0106-F	AGGAGAGGCTTTGTTTGGAGG	USP1	P0106-F
P0107-R	AACCACCGGACAAGCACAA	USP1	P0107-R
P0292-F	GCGAGTTGAAGAAGATCCA	WRKY40	P0292-F
P0295-R	TGTCACACATCAAGGTTAGCATC	WRKY40	P0295-R
P366-R	CTCAAGCACCATATACACTTCA	WRKY33	P366-R
P367-R	CCTTTGCTCTAGAGAATCCACC	WRKY33	P367-R

Abbreviations: F: forward; R: reverse.

## Data Availability

RNA-Seq data are available at NCBI’s Gene Expression Omnibus GEO Series under accession number GSE348911. Homozygous seeds of the *usp1-1* and *usp1-2* mutant lines are available from the corresponding authors upon request.

## Supplementary Materials

The following supporting information can be downloaded at https://www.mdpi.com/article/10.3390/ijms27219426/s1.
