# Supplementary material for: USP1, an MAPK Target with Chaperone-like Activity, Regulates Innate Plant Immunity in Arabidopsis
Source: Int J Mol Sci. 2026 Oct 22;27(21):9426. doi: 10.3390/ijms27219426 (PMC13762857; doi:10.3390/ijms27219426)
Supplement: Supplementary file 1 [file ijms-27-09426-s001.zip › ijms-4502369-supplementary.pdf]

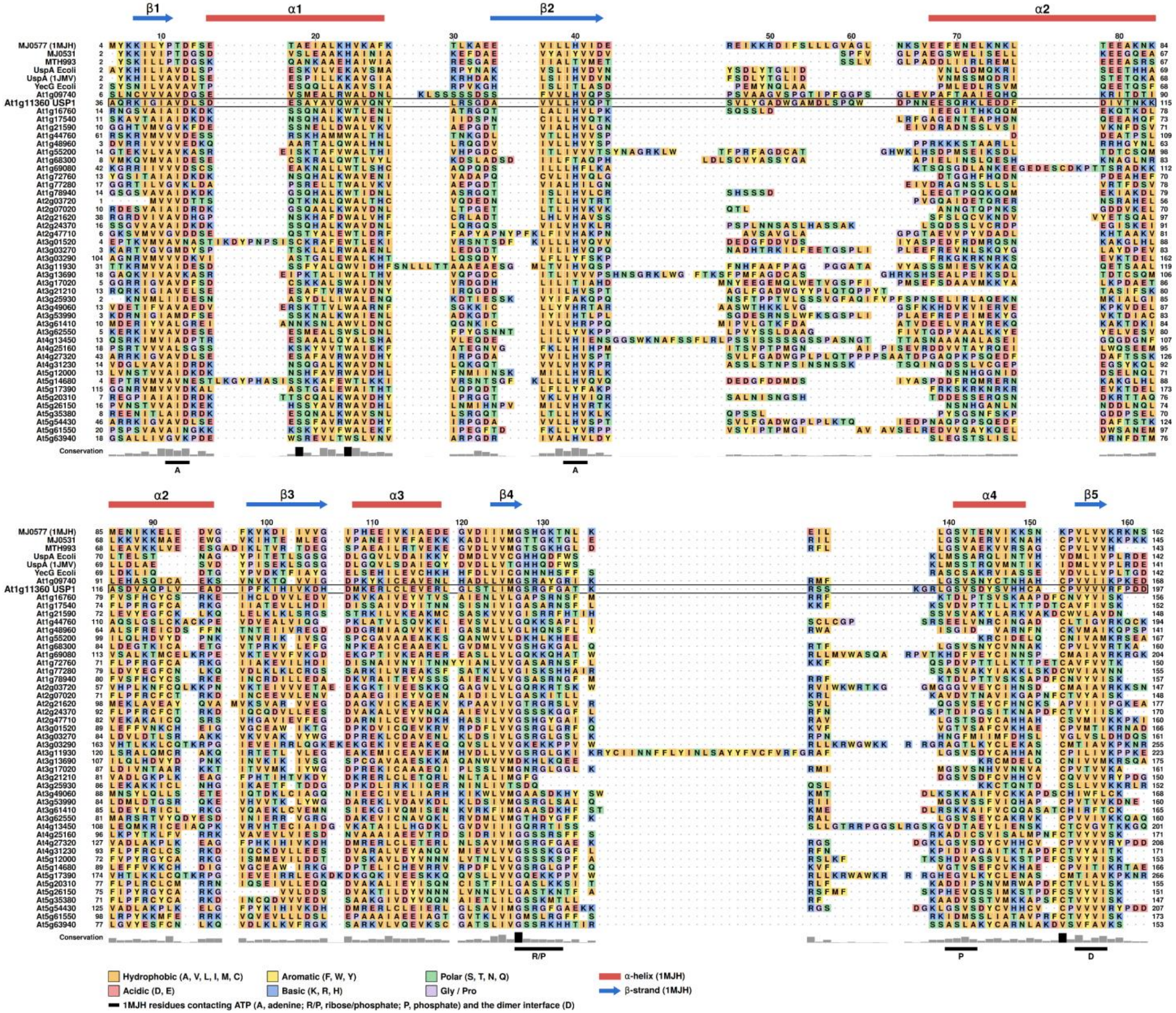

Figure S1. Structural conservation of the UspA domain across *Arabidopsis* and bacterial species. Multiple sequence alignment of the USP domain of 44 *Arabidopsis thaliana* USP proteins (Kerk et al., 2003) and six prokaryotic homologs: MJ0577 and MJ0531 (*Methanocaldococcus jannaschii*), MTH993 (*Methanothermobacter thermautotrophicus*), UspA and YecG (*Escherichia coli*) and UspA (*Haemophilus influenzae*, PDB 1JMV). All *Arabidopsis* domains align along the full length of the MJ0577 structure (PDB 1MJH; Zarembinski et al., 1998), whose secondary structure elements are shown above the alignment (α-helices, red; β-strands, blue). Black bars below mark the MJ0577 residues involved in ATP binding and dimerization, labeled as in Kerk et al. (2003): A, adenine; R/P and P, ribose and phosphate; D, dimerization region. USP1 (At1g11360, boxed) retains the ATP-binding motif G-x_2-G-x_9-G-(S/T) of MJ0577, which is absent from the non-ATP-binding *H. influenzae* UspA (Sousa and McKay, 2001). Residues are coloured by physicochemical class and dots indicate gaps. The conservation track shows the frequency of the most common residue per column (black, ≥ 80%). Modified from the Kerk et al. (2003).

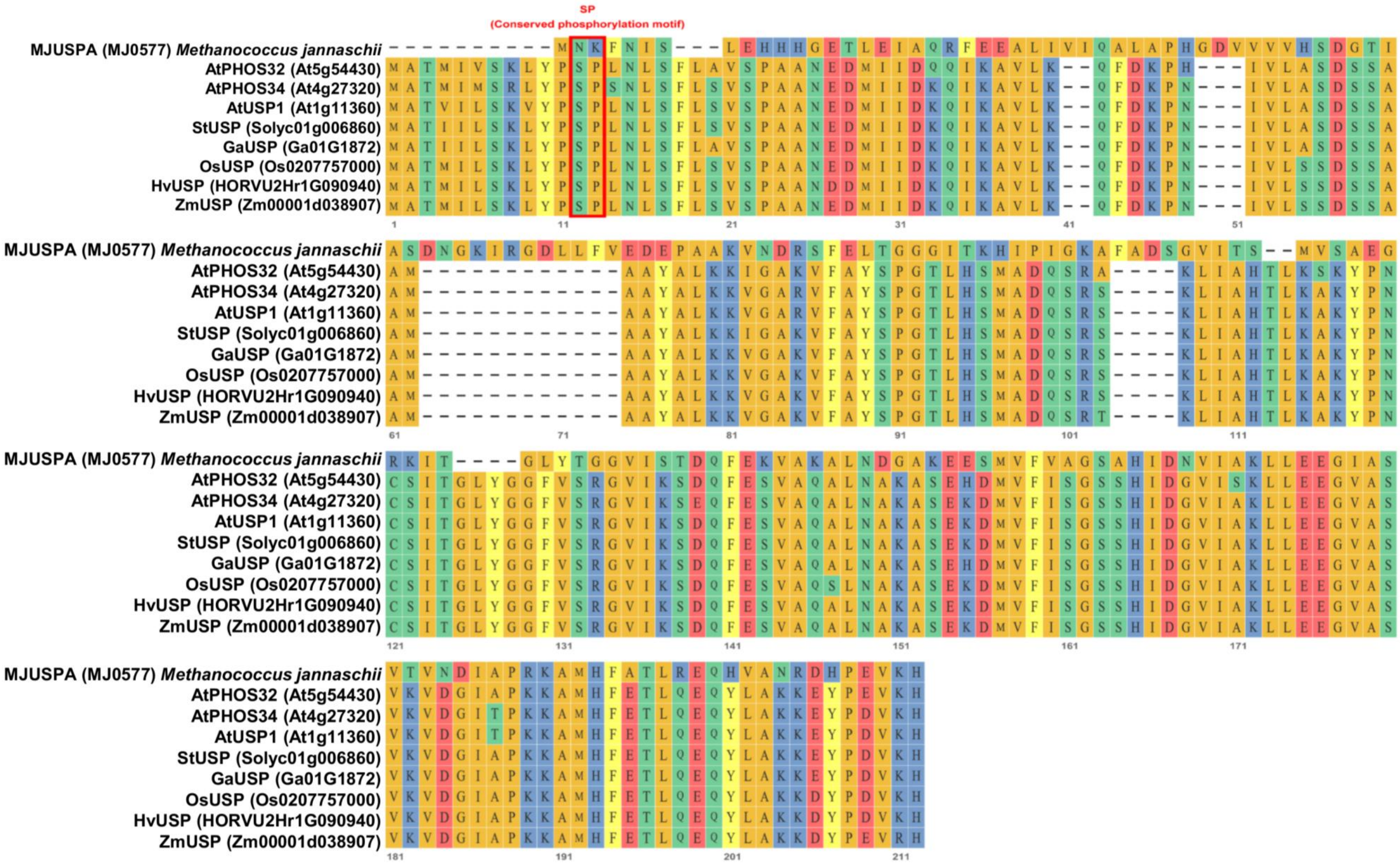

Figure S2. **Evolutionary conservation of the SP phosphorylation motif**. Multiple sequence alignment of USP homologs across plant species and archaea. Amino acid sequences of USP proteins from *Methanococcus jannaschii* (MJUSPA, MJ0577), *Arabidopsis thaliana* (AtPHOS32, At5g54430; AtPHOS34, At4g27320), *Solanum lycopersicum* (StUSP, Solyc01g006860), *Gossypium arboreum* (GaUSP, Ga01G1872), *Oryza sativa* (OsUSP, Os0207757000), *Hordeum vulgare* (HvUSP, HORVU2Hr1G090940), and *Zea mays* (ZmUSP, Zm00001d038907) were aligned using ClustalW. The conserved SP (Ser-Pro) phosphorylation motif is highlighted by a red box, representing the canonical MAPK phosphorylation consensus site. Residues are colour-coded by physicochemical properties: hydrophobic (orange/yellow), polar (green), charged (blue/magenta), and glycine/proline (pink). The alignment reveals high conservation of the SP motif from archaea (*M. jannaschii*) through dicots (*Arabidopsis*, *Gossypium*, *Solanum*) to monocots (*Oryza*, *Hordeum*, *Zea mays*), suggesting an evolutionarily conserved MAPK-mediated regulatory mechanism of USP proteins. Numbers below the alignment indicate amino acid positions.

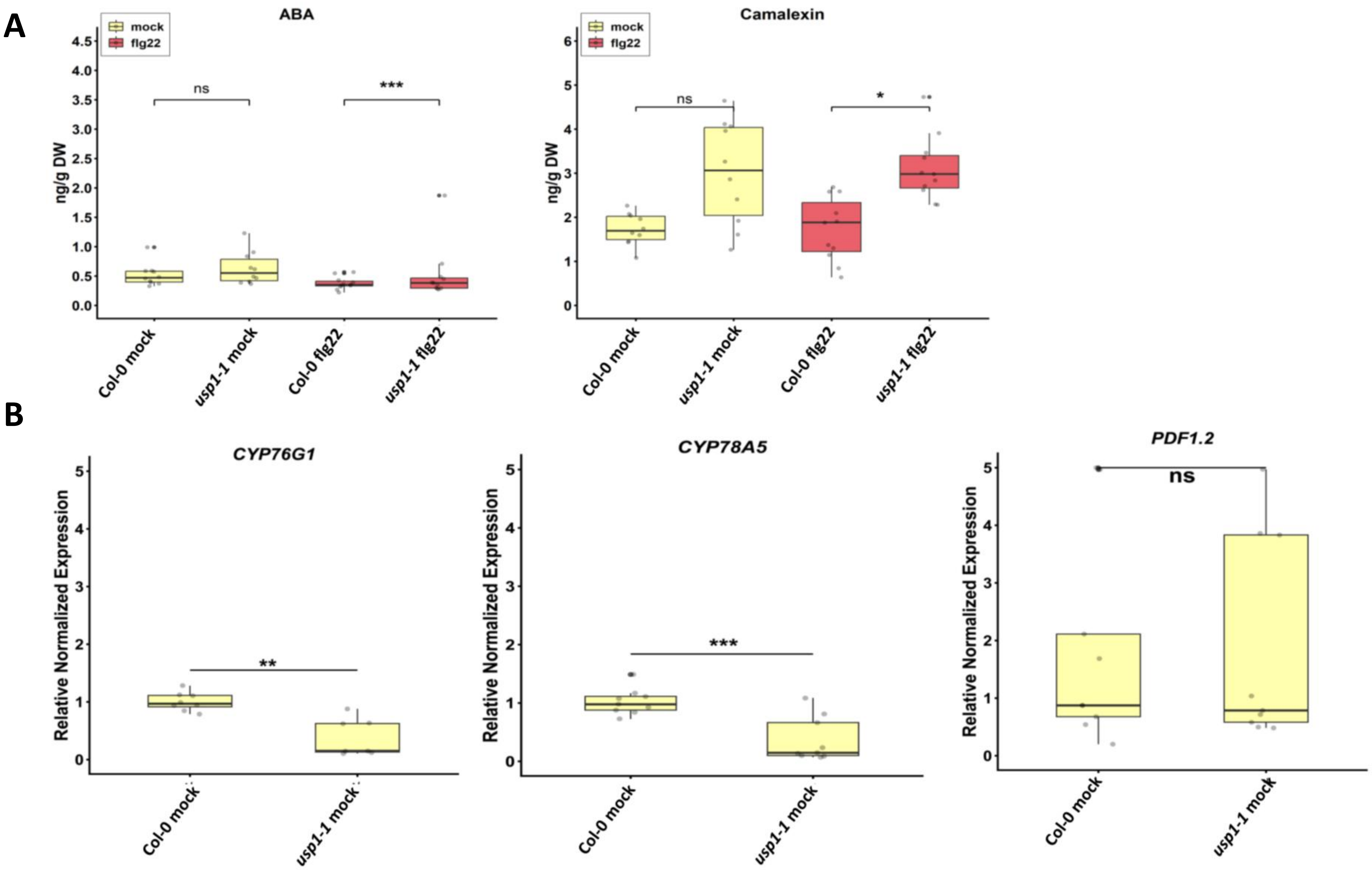

Figure S3. **ABA and Camalexin quantification and qRT-PCR validation**. **(A)** Quantification of ABA and camalexin levels in Col-0 (WT) and *usp1-1* mutant lines analysed by LC-MS/MS, with or without flg22 treatment for 1 hr. Three seedlings per tube (100 mg) and 18 plants per biological replicate for each line were used. Values are expressed as ng/g dry weight (DW). **(B)** qRT-PCR validation of *CYP76G1*, *CYP78A5*, and *PDF1.2* expression in wild-type and *usp1-1* plants under mock conditions. Expression was normalized to *UBQ(At4g05050)* and *ACTIN2(At3g18780)*. Vertical bars indicate the standard error for three independent biological experiments. The error bars represent SEM (standard error of mean); one asterisk (*) indicates P < 0.05, two (**) P < 0.01, three (***) P < 0.001; ns, not significant (Student's t test).

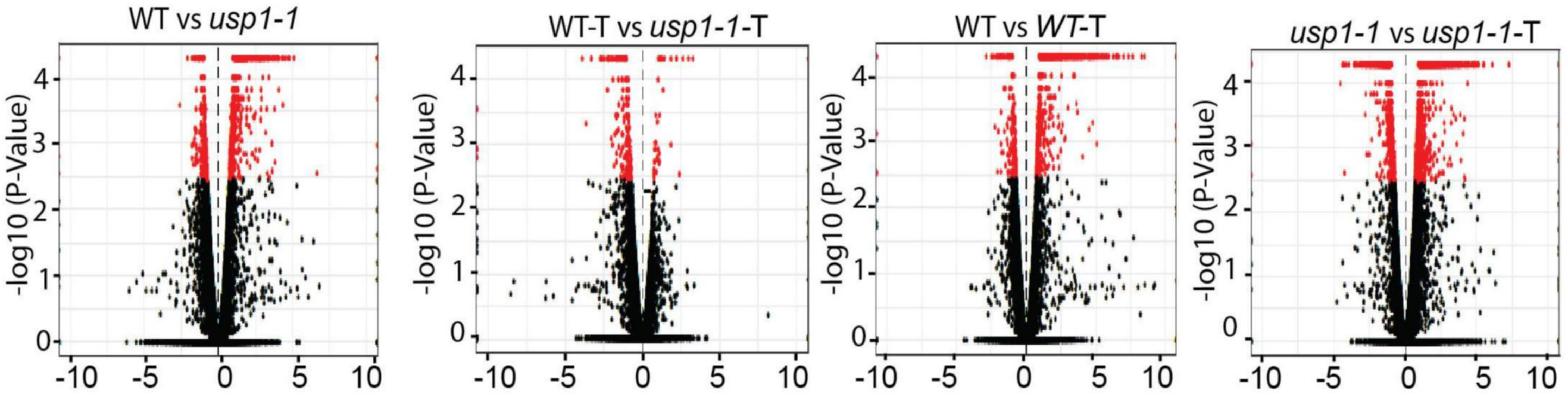

Figure. S4. Volcano plots of differential gene expression in response to flg22 treatment. volcano plots showing differential gene expression comparisons between mock and flg22-treated samples of WT(Col-0) and *usp1-1* mutant. The $\log_2$ fold change is plotted on the x-axis against the $-\log_{10}$(P-value) on the y-axis. Each plot represents a different comparison: WT mock vs. WT flg22, *usp1-1* mock vs. *usp1-1* flg22, and WT vs. *usp1-1* under flg22-treated conditions.

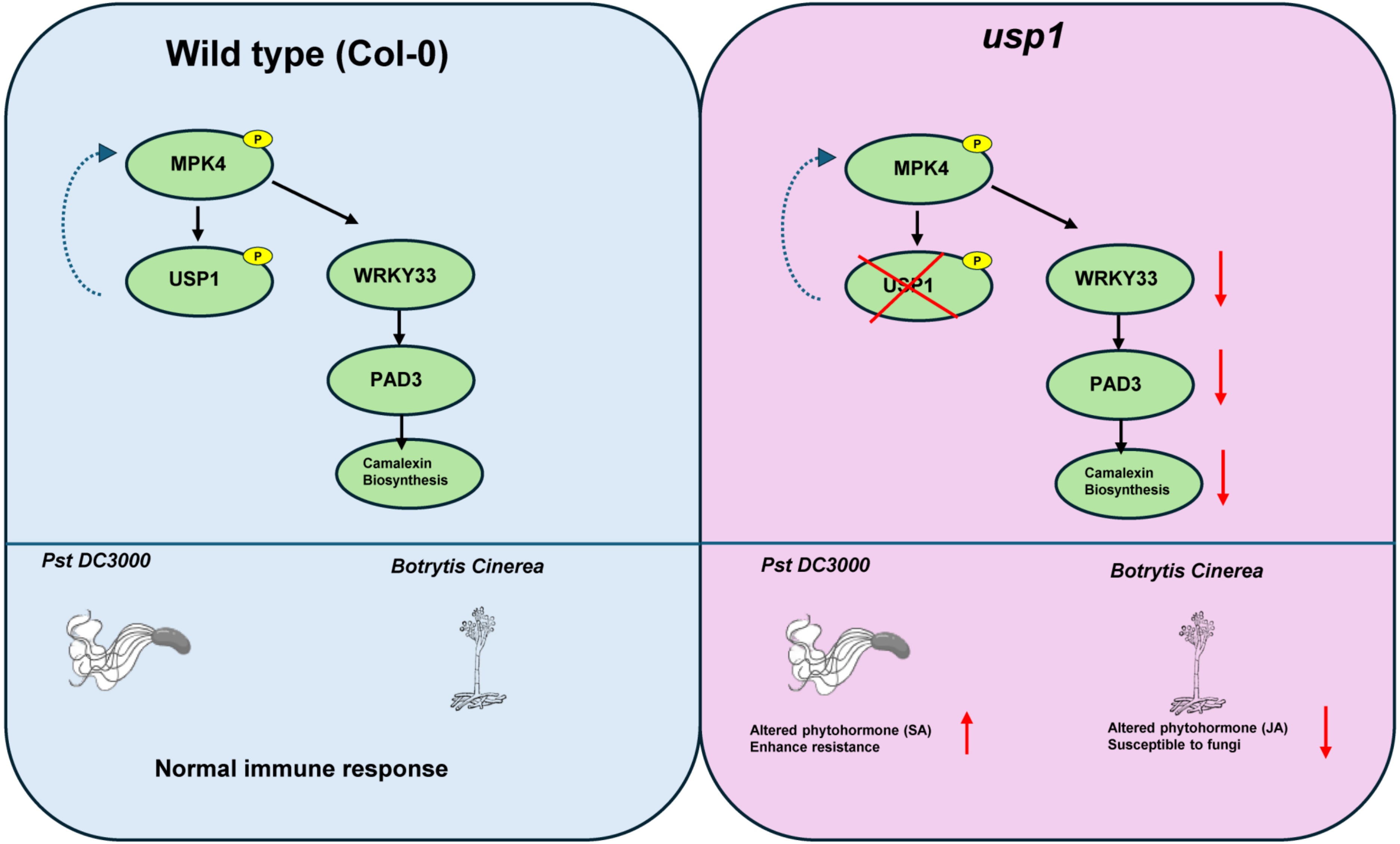

Figure. S5. A proposed working model for the role of USP1 in plant immunity. In wild type (Col-0), USP1 is phosphorylated by MPK3/4/6 upon MAMP perception, enhancing its molecular chaperone activity. Phosphorylated USP1 (USP1-P) stabilizes MPK4 protein, which activates the WRKY33–PAD3 camalexin biosynthesis pathway and maintains SA/JA hormone homeostasis (left panel). In the *usp1* mutant, loss of USP1 chaperone function leads to reduced MPK4 protein stability, diminished WRKY33 and PAD3 activity, and decreased camalexin biosynthesis. The resulting hormone imbalance elevated SA and reduced JA confers enhanced resistance to the biotrophic pathogen *Pseudomonas syringae* pv. tomato DC3000 but increased susceptibility to the necrotrophic fungus *Botrytis cinerea* (right panel). Solid arrows indicate activation or phosphorylation; dashed curved arrows indicate protein stabilization; red crosses denote loss of function; red arrows indicate increased (↑) or decreased (↓) levels. P, phosphorylation.

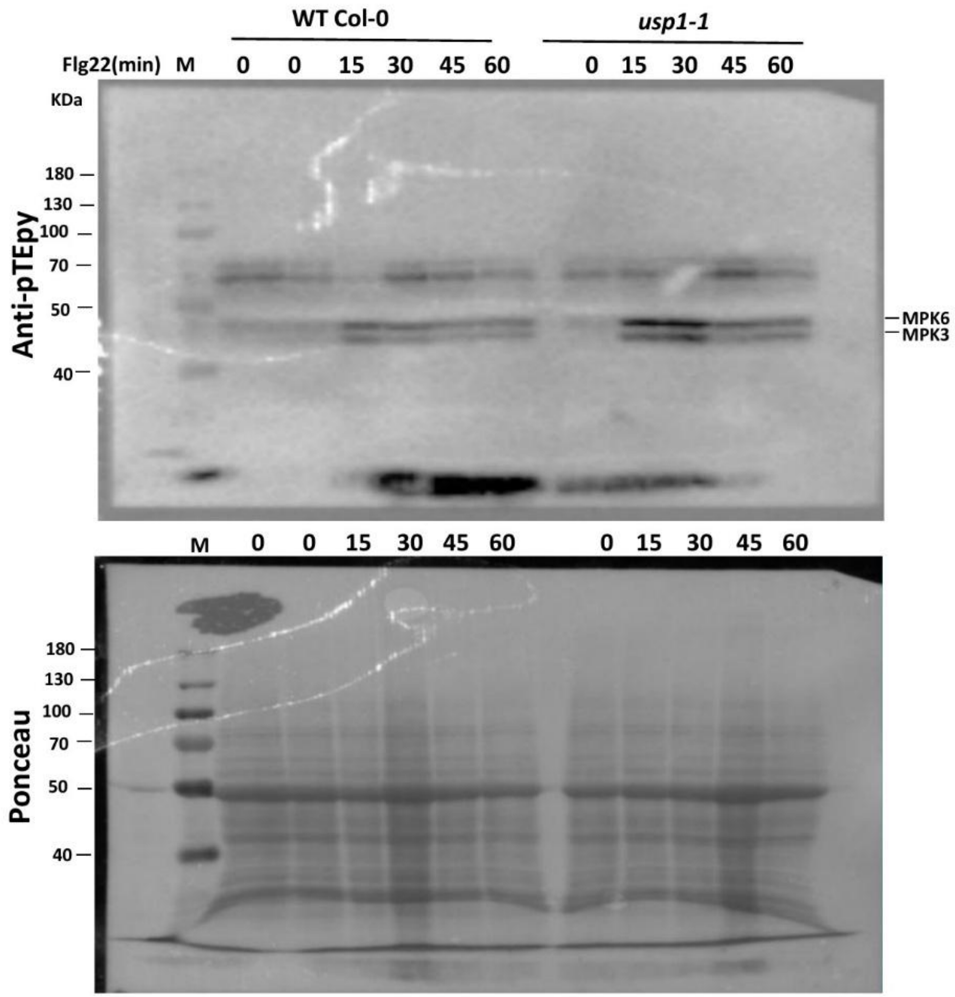

Figure S6. Original uncropped images for Figure 1C. Upper panel: immunoblot of total protein extracts from WT (Col-0) and *usp1-1* seedlings treated with 1 μM flg22 for the indicated times, probed with anti-phospho-p44/42 MAPK (anti-pTEpY) antibody. Lower panel: the same membrane stained with Ponceau S as a loading control. M, PageRuler Prestained Protein Ladder (10–180 kDa; Thermo Fisher Scientific, Waltham, MA, USA); marker sizes are given in kDa.
